# Necrotizing Pneumonia Secondary to Pulmonary Blastomycosis: A Case Report

**DOI:** 10.7759/cureus.38846

**Published:** 2023-05-10

**Authors:** Kyle A Burton, Matthew Karulf

**Affiliations:** 1 Internal Medicine, Michigan State University College of Human Medicine, Marquette, USA; 2 Pulmonology, Upper Peninsula Health Systems, Marquette, USA

**Keywords:** bronchoalveolar lavage, fungus, blastomyces, necrotizing pneumonia, pulmonary blastomycosis

## Abstract

Necrotizing pneumonia is a rare but potentially life-threatening complication of pulmonary blastomycosis, a fungal infection caused by inhaling spores of the fungus Blastomyces dermatitidis. This case report describes a 56-year-old male who presented with worsening malaise, subjective fevers and chills, night sweats, and a productive cough. Further evaluation revealed a right upper lobe necrotizing pneumonia secondary to pulmonary blastomycosis.

## Introduction

Blastomycosis is a fungal infection caused by Blastomyces dermatitidis, a fungus that is typically found in the soil of regions including the Ohio and Mississippi River Valleys, Great Lakes region, and the southeastern United States [1]. Pulmonary blastomycosis is an infection of the lungs caused by Blastomyces that often results in non-specific clinical symptoms such as fevers, chills, headache, cough, dyspnea, and chest pain [2]. We describe a patient presenting with subjective fevers, chills, night sweats, and a productive cough who was referred to pulmonology for evaluation of a right upper lobe opacity discovered on a chest X-ray. Further examination, including bronchoscopy and histologic examination, revealed the presence of Blastomyces, confirming the diagnosis of necrotizing pneumonia secondary to pulmonary blastomycosis.

## Case presentation

**Figure 1:**
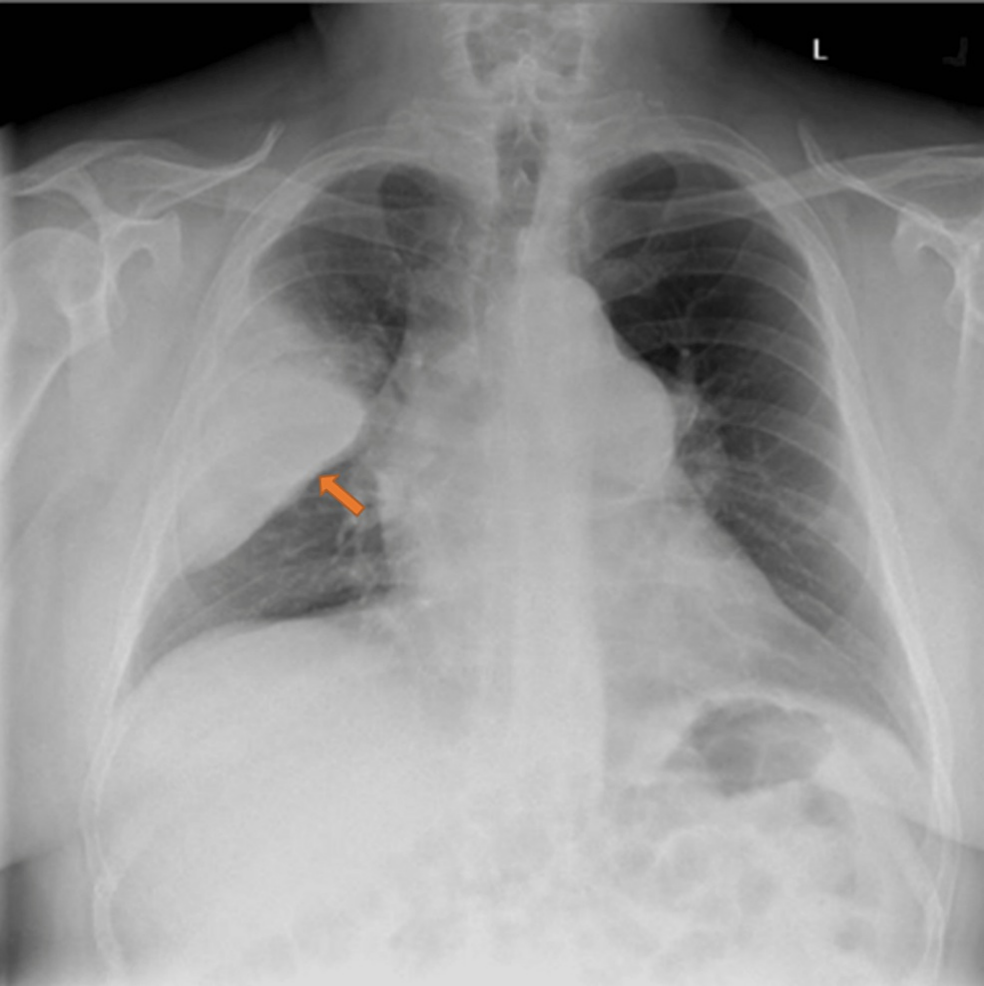
Initial chest X-ray on hospital admission showing right upper lobe opacity concerning for an infectious/inflammatory process versus malignancy (arrow).

**Figure 2:**
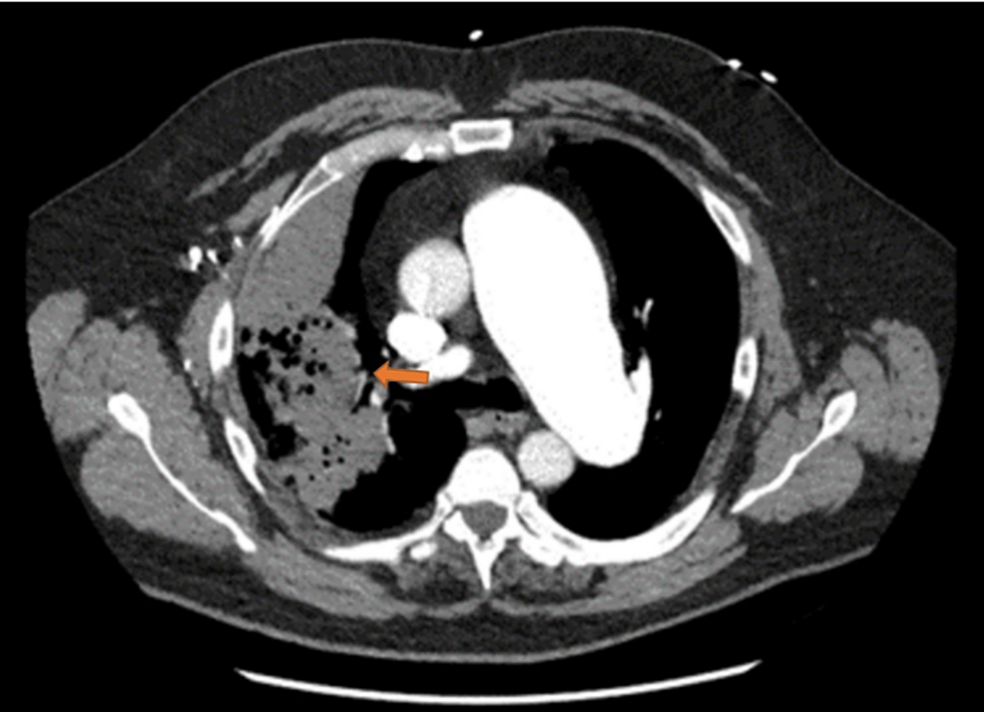
CT chest angiography showing right upper lobe necrotizing pneumonia (arrow).

A 56-year-old Caucasian male with a past medical history of a pulmonary artery aneurysm and hypertension was referred to pulmonology for bronchoscopy. This patient had presented to the emergency department with a one-month history of worsening malaise, subjective fevers, chills, night sweats, and a productive cough. Prior to presenting to the emergency department, he was treated with azithromycin and prednisone for these symptoms. On presentation to the emergency department, he had significant dyspnea and pleuritic chest pain prompting hospital admission. Labs at this time were notable for a white blood cell count of 18,700 white blood cells per microliter, negative influenza A, influenza B, SARS, and RSV antigens. Chest X-ray on admission showed a right upper lobe opacity concerning for an infectious/inflammatory process versus malignancy as shown in Figure 1. Follow-up computed tomography (CT) chest angiography showed an opacity of the right upper lobe measuring 16.5 × 9.5 × 8.8 cm in anteroposterior, transverse, and craniocaudal dimensions respectively as shown in Figure 2.

**Figure 3:**
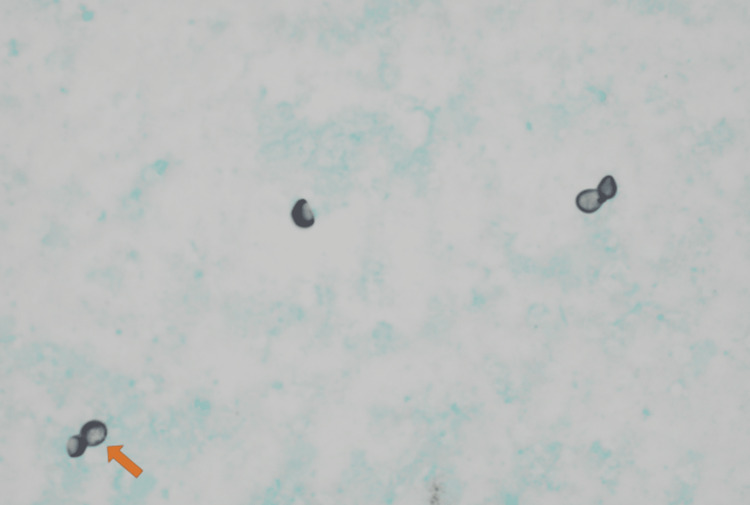
Grocott methenamine silver stain of sample collected via bronchoscopy showing large fungal spores with broad-based budding consistent with Blastomycosis (arrow).

Bronchoscopy with bronchoalveolar lavage and brushings were performed on the day of hospital admission. Mucopurulent secretions were noted in the right upper lobe without endobronchial lesions or active bleeding. Histologic examination of bronchial lavage and bronchial brushings revealed large fungal spores with broad-based budding on Grocott methenamine silver staining consistent with Blastomyces as shown in Figure 3. Serum Blastomyces antigen resulted positive at this time and bronchoalveolar lavage collections eventually grew Blastomyces dermatitidis on culture. Review of the patient’s history reveals that he may have been exposed to Blastomyces through his association with lumber manufacturing.

**Figure 4:**
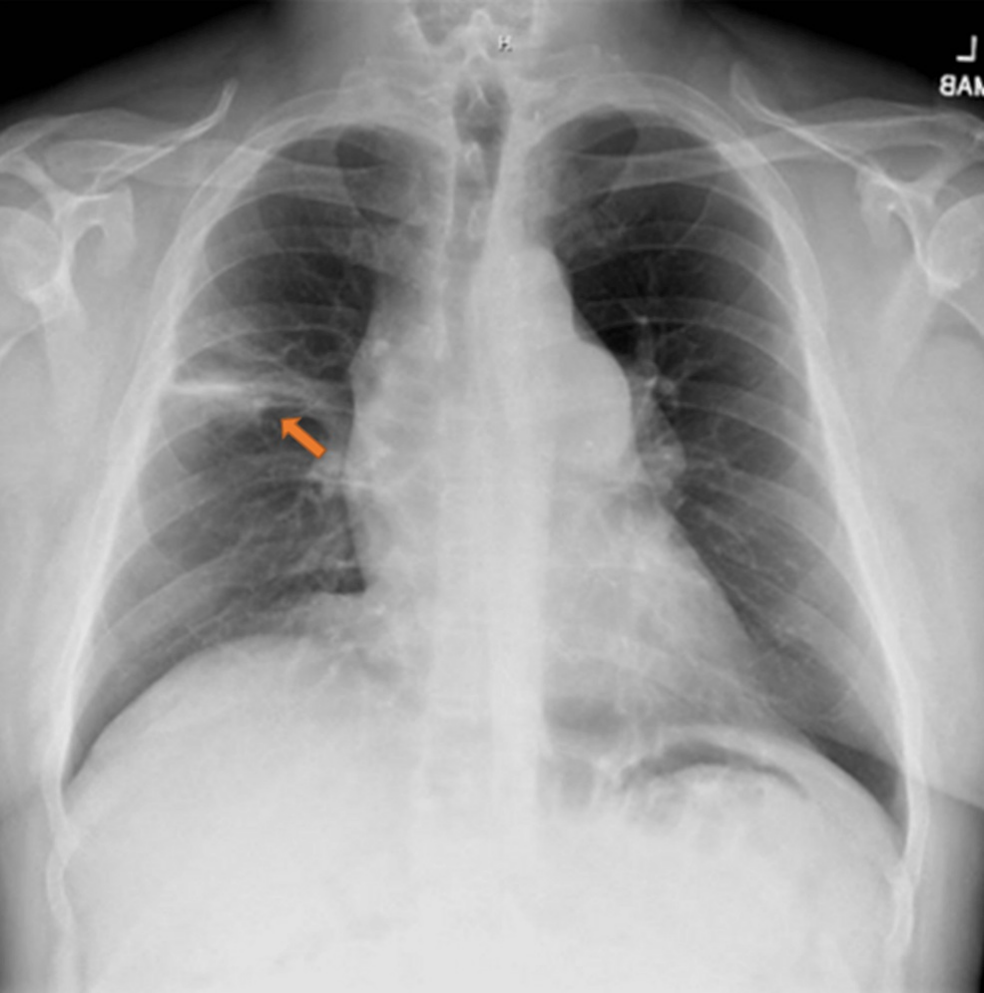
Repeat chest X-ray showing significantly improved right upper lobe consolidation as compared with initial chest X-ray from two months prior (arrow).

Given the evidence of blastomycosis, the patient was given 10 days of IV amphotericin 3 mg/kg with acetaminophen and diphenhydramine premedication. After completion, the patient was then started initially on itraconazole 200 mg by mouth three times daily for three days then 200 mg by mouth twice daily planned for 6-12 months. The patient was discharged from the hospital and he maintained follow-up with infectious disease. Serum itraconazole levels were found to be within the therapeutic range two weeks after beginning itraconazole. Two months after discharge, urine Blastomyces antigens were negative with no detectable antigens and chest X-ray revealed significantly improved right upper lobe consolidation as shown in Figure 4. After receiving four months of antifungal therapy with itraconazole, the patient desired to stop the medication due to persistent gastrointestinal discomfort. Itraconazole was discontinued and the patient was started on fluconazole 400 mg which was carried out for an additional two months.

## Discussion

Necrotizing pneumonia is a severe form of pneumonia with a high morbidity and mortality that often leads to pulmonary gangrene [3]. The most common causes of necrotizing pneumonia are Staphylococcus aureus and Streptococcus pneumoniae, with less common causes including Klebsiella and Haemophilus species and Pseudomonas aeruginosa [4]. CT chest is often the most sensitive diagnostic modality for diagnosing necrotizing pneumonia [5].

Pulmonary blastomycosis is a lung infection caused by the organism Blastomyces dermatitidis that often results in non-specific clinical symptoms such as fevers, chills, headache, cough, dyspnea, and chest pain [2]. Risk factors for the development of pulmonary blastomycosis include collagen vascular disease, outdoor occupations, and exposure to a coworker with blastomycosis [6]. Risk factors for the development of severe blastomycosis may additionally include immunosuppression, pulmonary multilobar disease, diabetes mellitus, and obesity [7]. In rare cases such as this one, pulmonary blastomycosis may result in a necrotizing pneumonia. Other cases have documented pulmonary blastomycosis resulting in the development of acute respiratory distress syndrome in addition to necrotizing pneumonia [8,9].

Diagnosis of pulmonary blastomycosis is supported by culture and visualization of multinucleated yeast with broad-based budding on histologic examination [10]. Itraconazole is the treatment of all presentations of blastomycosis including pulmonary blastomycosis. Liposomal amphotericin B can be used for severe blastomycosis infections [1]. In the setting of unsuccessful medical management, patients with necrotizing pneumonia may benefit from surgical intervention [11].

## Conclusions

Necrotizing pneumonia is a rare but serious complication of pulmonary blastomycosis, a fungal infection caused by Blastomyces dermatitidis. This case report highlights the importance of considering blastomycosis in the differential diagnosis of patients presenting with non-specific symptoms such as fevers, chills, night sweats, and a productive cough, particularly in areas where Blastomyces is endemic. Early diagnosis and prompt initiation of antifungal therapy are critical for the successful management of pulmonary blastomycosis, as demonstrated in this case report.
